# Today’s Problem Tomorrow’s Crisis: A Retrospective, Single-Centre Observational Study of Nonagenarians in the Emergency Department

**DOI:** 10.7759/cureus.73460

**Published:** 2024-11-11

**Authors:** Erdinç Senguldur, Kudret Selki

**Affiliations:** 1 Emergency Medicine, Duzce University School of Medicine, Düzce, TUR

**Keywords:** adult emergency department, elderly patients, emergency department management, nonagenarians, oldest old

## Abstract

Purpose

Our aim was to analyse the Emergency Department (ED) admissions of patients aged 90 years and older (nonagenarians) and to provide the literature with data showing the needs and characteristics of this highly vulnerable group in the ED.

Methods

This was a retrospective, single-centre observational study from Türkiye. A total of 18,225 patients aged 65 years and older, who were admitted to the ED between January 1, 2022, and December 31, 2023, were identified and included in the study. The characteristics of the long-livers and patients aged less than 90 years were compared with each other. Continuous data were compared between the two groups by the Mann-Whitney U test. The relationships between two categorical variables were analysed by Pearson's Chi-square test.

Results

The proportion of long-livers among elderly patients was 3.9% (n = 711). A total of 77.6% (n = 552) of the long-livers were admitted by ambulance. The 48-hour, 30-day, and 180-day mortality rates of long-livers were greater than those of elderly patients under 90 years of age: 1.4% (n = 10) vs. 0.4% (n = 69), 5.8% (n = 41) vs. 2% (n = 352), and 10.5% (n = 75) vs. 4.9% (n = 858). The most common diagnoses in the ED are falls, upper respiratory tract infections, and asthma/chronic obstructive pulmonary disease (COPD) attacks.

Conclusions

The mortality rate of long-livers is quite high, and the decision to discharge patients from the ED should be made with extreme caution. Precautions to be taken about falls may prevent injuries. The establishment of geriatric EDs, and the allocation of vehicles to be used for hospital admissions of long-livers, will reduce the development of ED crowding in the future.

## Introduction

The population of elderly people is increasing rapidly worldwide. Due to developments in the fields of technology and medicine, as well as good living conditions, ageing in society is becoming more noticeable, especially in socioeconomically developed Western countries [1,2]. In European Union countries, the population of people aged 65 years and over is expected to double in the next 40 years and reach approximately 148 million people. It is known that people aged 65 years and over constitute more than 20% of the population in some developed Western countries, and it is thought that this rate will increase in the following years [3,4].

It is known that elderly patients are admitted to the Emergency Department (ED) with more serious clinical presentations, and the development of adverse outcomes is more frequent. In addition, elderly patients are those whose diagnosis and treatment are difficult because they have many comorbid diseases, use many different drugs, present with atypical symptoms, and cannot express their complaints due to decreased cognitive function [3,5-7]. Difficulties in the management of elderly patients prolong their length of stay in the ED and cause ED overcrowding. The number of ED admissions by elderly patients is expected to double in the next 20 years, and the effect of the elderly population on ED crowding is predicted to increase over the years [2,8,9].

Elderly patients are grouped as youngest old (aged 65-74 years), middle old (aged 75-89 years), and oldest old or nonagenarians (aged 90 years and older) [1]. It is predicted that the proportion of nonagenarians in the elderly population will increase in the following years [10]. Reasons such as decreased physical abilities, high rates of dementia, chronic diseases, and generally needing care indicate that nonagenarians will cause more burden on social services and health systems in terms of labour force and costs in the following years.

In most of the previous studies on ED admissions of elderly patients, patients aged 65 years and older were analysed. In studies conducted in this way, age groups with different needs and complaints are evaluated together, and, in our opinion, accurate results cannot be obtained. The aim of this study was to analyse the ED admissions of patients aged 90 years and over and to provide the literature with data showing the needs and characteristics of this highly vulnerable group in the ED, whose proportion is increasing in society.

This article was previously posted to the Research Square preprint server on May 15, 2024.

## Materials and methods

Study setting and design

This was a retrospective, single-centre observational study. It was conducted in the ED of a tertiary university hospital in Türkiye, with approximately 100,000 admissions per year. The study was initiated after local ethics committee approval (approval ID: 2024/49, dated March 4, 2024) was obtained. Between January 1, 2022, and December 31, 2023, the study included patients aged 65 years and older who were admitted to the ED, and patients aged 90 years and older selected from this group.

Patients aged 65 years and older, admitted to the ED during the specified period, were identified through the hospital computer system and included in the study. Age, gender, hemogram results, emergency blood biochemistry, and mortality data for 48 hours, 30 days, and 180 days were gathered from the hospital's records. For patients aged 90 years and over, information on comorbid diseases, time of admission, hospitalisation status, and admission diagnosis were also noted.

Selection of participants and study protocol

The inclusion criteria were being 65 years of age or older, and being admitted to the ED. Patients with incomplete data were excluded from the study. Patients were divided into three shift groups according to the time of presentation. Such a grouping was made considering that these three different shift groups - working hours, evening hours, and night hours - will have different patient admission profiles. Those who presented between 00:00 and 07:59 were grouped as Shift 1; those who presented between 08:00 and 15:59 were grouped as Shift 2; and those who presented between 16:00 and 23:59 were grouped as Shift 3. The time interval defined as Shift 2 in the study is referred to as in-hours in Türkiye, the country where the study was conducted.

Descriptive statistical data of the patients included in the study were generated in terms of the scanned parameters. Patient groups aged 90 years and older, and patients aged 90 years and younger, were compared with each other in terms of the characteristics screened in the study. Nonagenarians were analysed for comorbid diseases and diagnoses made in the ED. Shift groups, formed according to the time of admission, were compared in terms of age, gender, hospitalisation, and 48-hour, 30-day, and 180-day mortality.

Data analysis

Continuous data were summarised as median, 25th and 75th percentiles, and categorical data were summarised as frequency and percentage. Compliance with a normal distribution was evaluated by the Shapiro-Wilk test, Kolmogorov-Smirnov test, and histogram analysis. The continuous data screened in the study were not normally distributed. Continuous data were compared between two groups by the Mann-Whitney U test. The relationships between two categorical variables were analysed by Pearson's Chi-square test or Fisher's exact test. IBM SPSS Statistics for Windows, Version 23 (Released 2015; IBM Corp., Armonk, NY, USA) was used for these analyses. The significance level was set as p < 0.05.

## Results

Among a total of 119,259 ED visits made during the study period, the number of visits made by patients aged 65 years and over, whose data could be accessed, was 18,225. It was observed that 711 of these visits were made by nonagenarians. In this study, 15.3% of the patients in the ED were aged 65 years and older. The percentage of nonagenarians was 3.9% among patients aged 65 years and over, and 0.06% among all emergency patients.

The median age of the patients aged 65 years and older was 73 (68-70) years, and 49.9% (n = 9101) were female. A total of 16.0% (n = 2907) of patients aged 65 years and older were hospitalised. A total of 0.4% (n = 79) of the patients aged 65 years and older died within 48 hours, 2.2% (n = 393) died within 30 days, and 5.1% (n = 933) died within 180 days. In the group of nonagenarians, the female sex ratio was greater than that in the group of patients younger than 90 years (60.2% (n = 428) vs. 49.5% (n = 8673); p < 0.001). Nonagenarians had a higher 48-hour mortality rate (1.4% (n = 10) vs. 0.4% (n = 69); p < 0.001), 30-day mortality rate (5.8% (n = 41) vs. 2% (n = 352); p < 0.001), and 180-day mortality rate (10.5% (n = 75) vs. 4.9% (n = 858); p < 0.001) than patients aged <90 years. The characteristics of patients aged 65 years and older in terms of the parameters screened in the study, and the comparison of patients aged <90 years and nonagenarians in terms of the parameters screened in the study, are shown in Table 1.

**Table 1:** Comparison of nonagenarians with patients under 90 years in terms of age, gender, lab results, hospitalisation, and mortality. Bold p-values indicate a statistically significant difference between the groups (p < 0.05). Data were presented as n(%) or median (interquartile range). The Chi-square test was used for comparisons made with categorical data. The Mann-Whitney U test was used for comparisons with continuous data.

Parameter	Reference rates	≥65 years (n = 18,224)	≥65 to <90 years (n = 17,513)	≥90 years old (n = 711)	p-value
Gender, female	-	9101 (49.9%)	8673 (49.5%)	428 (60.2%)	<0.001
Haemoglobin (g/dL)	11.00-15.50	11.93 (10.50-13.18)	11.96 (10.52-13.21)	11.36 (10.18-12.60)	<0.001
Leukocyte (10^3/mm^3^)	4.00-10.60	8.03 (6.20-10.62)	8.02 (6.19-10.62)	8.17 (6.47-10.68)	0.138
Blood urea nitrogen (mg/dL)	15.00-50.00	20.05 (15.38-27.81)	19.83 (15.29-27.38)	25.29 (19.02-37.94)	<0.001
Creatinine (mg/dL)	0.70-1.20	1.02 (0.82-1.32)	1.01 (0.82-1.31)	1.13 (0.84-1.52)	<0.001
Aspartate transaminase (IU/L)	5-40	14.00 (10.00-20.00)	14.00 (10.00-20.00)	11.50 (8.22-17.00)	<0.001
Alanine transaminase (IU/L)	5-40	20.70 (16.00-28.00)	20.70 (16.00-28.00)	20.10 (16.00-28.80)	0.953
Total bilirubin (mg/dL)	0.25-1.20	0.50 (0.34-0.77)	0.50 (0.34-0.76)	0.52 (0.36-0.79)	0.020
Direct bilirubin (mg/dL)	0-0.20	0.18 (0.12-0.29)	0.18 (0.12-0.29)	0.20 (0.13-0.33)	<0.001
Sodium (mEq/L)	135-145	138 (135-140)	138 (135-140)	138 (136-140)	0.625
Potassium (mEq/L)	3.50-5.50	4.41 (4.05-4.80)	4.41 (4.06-4.80)	4.38 (3.95-4.77)	0.060
Chloride (mEq/L)	96-107	102 (99-105)	102 (99-105)	103 (100-106)	<0.001
Calcium (mg/dL)	8.60-1.20	9.20 (8.80-9.50)	9.20 (8.80-9.50)	9.00 (8.60-9.30)	<0.001
Uric acid (mg/dL)	2.00-7.00	5.50 (4.40-6.90)	5.50 (4.30-6.90)	6.00 (4.70-7.70)	<0.001
Albumin (g/dL)	3.50-5.20	4.05 (3.69-4.33)	4.07 (3.71-4.34)	3.82 (3.39-4.10)	<0.001
Hospitalisation (yes)	-	2907 (16%)	2777 (15.9%)	130 (18.3%)	0.083
Mortality in 48 hours	-	79 (0.4%)	69 (0.4%)	10 (1.4%)	<0.001
Mortality in 30 days	-	393 (2.2%)	352 (2%)	41 (5.8%)	<0.001
Mortality in 180 days	-	933 (5.1%)	858 (4.9%)	75 (10.5%)	<0.001

It was found that 77.6% (n = 552) of nonagenarians were admitted to the ED by ambulance. Additionally, 14.5% (n = 103) of the nonagenarians were admitted during Shift 1, 46% (n = 327) were admitted during Shift 2, and 39.5% were admitted during Shift 3. Among the nonagenarians, 91.1% (n = 648) had at least one comorbid disease. The most common comorbid diseases were hypertension (57.0% (n = 405)), cardiovascular diseases (41.2% (n = 293)), and dementia (39.8% (n = 283)). The prevalence of comorbid diseases among nonagenarians is shown in Table 2.

**Table 2:** Comorbid diseases of nonagenarians admitted to the Emergency Department.

Comorbidities	n (%)
Hypertension	405 (57.0%)
Cardiovascular system disorders	293 (41.2%)
Dementia	283 (39.8%)
Asthma/chronic obstructive lung disease	122 (17.2%)
Diabetes mellitus	103 (14.5%)
Benign prostatic hypertrophy	79 (11.1%)
Malignities	55 (7.7%)
Stroke	51 (7.2%)
Renal failure	24 (3.4%)

When the diagnoses of nonagenarians admitted to the ED were analysed, the most common diagnosis was falls (11.1% (n = 79)). Other common diagnoses were upper respiratory tract infections (10.4% (n = 74)), asthma or chronic obstructive pulmonary disease (COPD) attacks (10.4% (n = 74)), and pneumonia (8.3% (n = 59)). The diagnoses of nonagenarians who were admitted to the ED are shown in Table 3.

**Table 3:** Final diagnoses of nonagenarians in the Emergency Department.

Diagnose	n (%)
Falls	79 (11.1%)
Upper respiratory tract infection	74 (10.4%)
Asthma/chronic obstructive lung disease attack	74 (10.4%)
Pneumonia	59 (8.3%)
Oral intake disorder	39 (5.5%)
Myalgia	36 (5.1%)
Stroke	32 (4.5%)
Urinary tract infections	31 (4.4%)
Lung oedema	27 (3.8%)
Haematuria	21 (3.0%)
Gastroenteritis	19 (2.7%)
Gastrointestinal tract haemorrhage	17 (2.4%)
Hypertension	16 (2.3%)
Dyspepsia	15 (2.1%)
Soft tissue infection	15 (2.1%)
Biliary tract pathologies	12 (1.7%)
Constipation	8 (1.1%)
Myocardial infarction	8 (1.1%)
Cardiac arrest	7 (1.0%)
Other	122 (17.0%)

There was no statistically significant difference in age, gender, hospitalisation status, or 48-hour, 30-day, and 180-day mortality rates between the three shift groups formed according to the admission hours of nonagenarians (p > 0.016). The comparison between shift groups is shown in Table 4.

**Table 4:** Comparison of shift groups formed according to admission hours in terms of age, gender, hospitalisation, and mortality. Since the comparison was made between three groups, Bonferroni correction was applied. A p-value of <0.016 indicates a statistically significant difference between the groups. Data were presented as n(%) or median (interquartile range). The Chi-square test was used for comparisons made with categorical data, and the Mann-Whitney U test was used for comparisons with continuous data.

Parameter	Shift 1 (00:00-07:59) (n = 103)	Shift 2 (08:00-15:59) (n = 327)	Shift 3 (16:00-23:59) (n = 281)	p-value
Age, years	91 (90-94)	91 (90-94)	91 (90-93)	0.509
Gender, female	57 (55.3%)	204 (62.4%)	167 (59.4%)	0.420
Hospitalisation	19 (18.4%)	69 (21.1%)	42 (14.9%)	0.147
Mortality in 48 hours	2 (1.9%)	4 (1.2%)	4 (1.4%)	0.864
Mortality in 30 days	5 (4.9%)	21 (6.4%)	15 (5.3%)	0.774
Mortality in 180 days	9 (8.7%)	38 (11.6%)	28 (10%)	0.651

## Discussion

An analysis of the literature revealed that 12%-24% of ED admissions are made by patients 65 years of age or older. This rate is higher in socioeconomically developed countries, where life expectancy is higher [5,8-13]. The proportion of nonagenarians among elderly patients admitted to the ED also varies between societies. In a study conducted in Switzerland, the percentage of nonagenarians among elderly patients admitted to the ED was 24%; in a study conducted in Italy, it was 19%; and in a study conducted in the UK, it was approximately 30% [3,6,14]. In a study conducted in Australia, it was shown that 7.6% of the population aged 65 years and over consisted of people aged 90 years and over. In a study conducted in Türkiye, 10% of the patients who were admitted to the ED were aged 65 years and older, and 7.2% of these patients were aged 85 years and older [15]. The rate of approximately 15% of ED patients aged 65 years and over in our study is higher than that in the other study conducted in Türkiye and is compatible with the literature. The percentage of nonagenarians in elderly patients, which was 3.9% in our study, is lower than that in studies conducted in Western countries. In the other study conducted in Türkiye, it was thought that the percentage of nonagenarians was greater than that found in our study because nonagenarians were defined as patients aged 85 years and older. The difference from the studies conducted in European countries can be attributed to the fact that life expectancy in these societies is greater than that in Türkiye, due to their high socioeconomic status [16].

In ED patients aged 65 years and older, the female sex ratio has been shown to be between 50% and 52%. In nonagenarians, the female sex ratio increases to 60%-68% [2,6,14,17]. In our study, the female sex ratio of 60% in the nonagenarian group was similar to that in the literature. The high female sex ratio in the nonagenarian group may be explained by the fact that life expectancy is greater in women than in men. The fact that harmful habits, such as tobacco, alcohol, or substance use, are more common in men; that men are more exposed to trauma as a result of occupational reasons, wars, or accidents; and that diseases, which are the main causes of death, are more common in men may explain the shorter life expectancy of men compared to women [18].

The fact that the relatives of nonagenarians are generally elderly people is a factor that makes it difficult for them to access health services. It is very difficult for nonagenarians who are bedridden due to stroke or dementia to be transported to outpatient clinics or emergency services by their relatives. In previous studies, it was observed that 60%-70% of nonagenarians were transported to the ED by ambulance [6,19]. In our study, the rate of admission by ambulance was 77.6% (n = 552). The high rate of admission by ambulance may be explained by the easy access to ambulance services in Türkiye and the fact that ambulance requests made through the 112 emergency call lines almost always receive a positive response. Although easy access to ambulances and emergency health services is often abused, it cannot be ignored that it improves the quality of life for groups such as nonagenarians who need continuous support. Establishing vehicles other than general ambulances, which provide access to health services specifically for nonagenarian patients, will prevent the unnecessary occupation of ambulances. Examination and treatment areas - in other words, geriatric EDs - which will be established for elderly patients outside the EDs and provide 24-hour service, will prevent frequent ED visits, especially by nonagenarians, and help reduce ED crowding.

In this study, nonagenarians were mostly admitted during out-of-hours. The reason for this may be that nonagenarians are too old to apply to the hospital on their own, and their relatives cannot accompany them during these in-hours because they have to work. The fact that there was no difference between the shift groups in terms of hospitalisation or mortality rates suggests that the admission times were determined according to the availability of relatives, rather than the clinical conditions of the patients.

One of the main reasons that elderly patients are difficult to manage and are complicated patients in the ED is the presence of multiple comorbid diseases. Studies conducted with patients aged 65 years and older admitted to the ED have shown that 87%-97% of these patients have a comorbid disease [17,20]. Cardiovascular diseases, dementia, and COPD are the most common comorbid diseases in nonagenarians [3]. In this study, hypertension, cardiovascular disease, and dementia were found to be the most common comorbid diseases in nonagenarians. This study supports the findings in the literature, with a comorbidity rate of 91%. The incidence of hypertension increases with age. While hypertension is observed in approximately 29% of adults in the United States of America (USA), this rate increases to 65% in those aged 60 years and older [19]. Hypertension is one of the leading risk factors for cardiovascular diseases [21-23]. The increase in hypertensive years with advancing age may explain the frequency of cardiovascular diseases in nonagenarians. Hypertension is also a risk factor for the development of dementia [23]. Improving blood pressure control in nonagenarians is important for improving quality of life and reducing the development of comorbid diseases.

The incidence of dementia is gradually increasing in society, and its importance as a public health problem is becoming more evident. It is thought that 7.7 million people are diagnosed with dementia annually worldwide [23]. According to World Health Organization (WHO) data, there were 35.6 million dementia patients worldwide in 2010, and this number is expected to triple by 2050 [22]. Alzheimer's disease, dementia with Lewy bodies, and dementia due to Parkinson's disease - which are the most common causes of dementia - are common diseases of advanced age, especially in nonagenarians [24,25]. The increase in life expectancy indicates that the burden of dementia on healthcare systems will rise in the coming years. It is important to identify the factors that contribute to the development of dementia and reduce its effects, but we will encounter many more dementia patients in the coming years than today. Starting to organise centres where the care and treatment of dementia patients can be carried out together today has the potential to prevent possible crises in the healthcare system in the future.

According to the literature on ED diagnoses in nonagenarians, heart failure, trauma, pneumonia, and various infections constitute the main diagnoses [2,5,15,19]. In this study, the most common diagnosis was musculoskeletal injuries due to falls. Nonagenarians are prone to falls because of their reduced physical capacity. Due to the high incidence of dementia, nonagenarians may not be able to avoid behaviours that could cause falls and may not be able to protect themselves. People who care for nonagenarians should be cautious about the risk of falls and should avoid leaving the patient alone as much as possible during activities such as walking or climbing stairs. In toilets and bathrooms, which are places where falls frequently occur, it will be useful to install devices that nonagenarians can hold onto while moving. Another precaution that can be taken is to keep the toilets and bathrooms used by nonagenarians dry and clean to prevent slipping and falling. Upper respiratory tract infections and pneumonias were also among the most common diagnoses in our study. It is important for nonagenarians, whose immune systems are not as strong as those of younger people, to use masks that cover the mouth and nose when entering crowded areas. People known to have respiratory tract infections should avoid being in the same environment as nonagenarians, whenever possible, to protect them from infectious diseases. Exacerbations of chronic diseases such as COPD or heart failure were also common diagnoses in our study. Failure of nonagenarians to take their prescribed medication for these diseases as recommended will lead to the worsening of symptoms. It is important that those caring for nonagenarians check that patients are taking their medication regularly and at the appropriate dose.

In studies in which ED admissions of elderly patients were analysed, 0.6%-4% of patients in the nonagenarian age group were reported to die in the ED [3,6,14,19]. The 30-day mortality of nonagenarians after ED presentation was shown to be 8% in one study [16]. In our study, the 48-hour mortality rate of the nonagenarians after ED admission was 1.4%, which was similar to the in-ED mortality rates reported in previous studies. Advanced age is known to be a risk factor for mortality alone [10]. In this study, the fact that the 48-hour, 30-day, and 180-day mortality rates of the nonagenarian group were greater than those of the relatively young elderly may be explained in this way. Importantly, nonagenarians who are observed to have a high mortality rate should be evaluated in detail at ED admission. Prolonged waiting times, which may cause them to leave without being evaluated by a physician, should be avoided, and the treatments to be recommended at the end of the evaluation should be explained in detail to the patient and the people who provide care.

The first limitation of this study is that it was retrospective. The second limitation is that it was a single-centre study. Third, we did not have diagnostic or comorbid disease data for patients under the age of 90 years, so no comparison could be made.

## Conclusions

The mortality rate of nonagenarians is quite high, and the decision to discharge patients from the ED should be made with extreme caution. Waiting times that may cause nonagenarians to leave without being seen by a physician should be shortened. The prescribed treatments should be explained in detail to nonagenarians and their relatives, and readmissions due to exacerbations of chronic diseases should be prevented. Precautions to be taken regarding falls may prevent injuries. Providing special service vehicles that nonagenarians can use, instead of ambulances, for ED or polyclinic admissions will be beneficial for the efficient use of existing ambulances. Special examination and treatment areas, or geriatric EDs, to be established for nonagenarians will prevent ED crowding that may develop due to recurrent ED admissions of these patients, which are expected to increase proportionally in the future.
